# Matrix Metalloproteinase-9 Leads to Claudin-5 Degradation via the NF-κB Pathway in BALB/c Mice with Eosinophilic Meningoencephalitis Caused by *Angiostrongylus cantonensis*

**DOI:** 10.1371/journal.pone.0053370

**Published:** 2013-03-07

**Authors:** Ping-Sung Chiu, Shih-Chan Lai

**Affiliations:** 1 Institute of Medicine, Chung Shan Medical University, Taichung, Taiwan; 2 Department of Parasitology, Chung Shan Medical University, Taichung, Taiwan; 3 Clinical Laboratory, Chung Shan Medical University Hospital, Taichung, Taiwan; Institut national de la santé et de la recherche médicale - Institut Cochin, France

## Abstract

The epithelial barrier regulates the movement of ions, macromolecules, immune cells and pathogens. The objective of this study was to investigate the role of the matrix metalloproteinase (MMP)-9 in the degradation of tight junction protein during infection with rat nematode lungworm *Angiostrongylus cantonensis*. The results showed that phosphorylation of IκB and NF-κB was increased in mice with eosinophilic meningoencephalitis. Treatment with MG132 reduced the phosphorylation of NF-κB and the activity of MMP-9, indicating upregulation of MMP-9 through the NF-κB signaling pathway. Claudin-5 was reduced in the brain but elevated in the cerebrospinal fluid (CSF), implying that *A. cantonensis* infection caused tight junction breakdown and led to claudin-5 release into the CSF. Degradation of claudin-5 coincided with alteration of the blood-CSF barrier permeability and treatment with the MMP inhibitor GM6001 attenuated the degradation of claudin-5. These results suggested that degradation of claudin-5 was caused by MMP-9 in angiostrongyliasis meningoencephalitis. Claudin-5 could be used for the pathophysiologic evaluation of the blood-CSF barrier breakdown and tight junction disruption after infection with *A. cantonensis*.

## Introduction

The rat nematode lungworm *Angiostrongylus cantonensis* undergoes obligatory intracerebral migration in its hosts and induces the parasitic disease angiostrongyliasis [1]. Infection with this parasite induces severe central nervous system (CNS) disease, especially eosinophilic meningitis [2] or meningoencephalitis [3] in non-permissive hosts (human or mice). Matrix metalloproteinase (MMP)-9 activity is closely associated with angiostrongyliasis meningitis caused by infection with *A. cantonensis* [4], [5]. This enzyme is associated with disruption of the blood-CNS barrier in mice with angiostrongyliasis meningitis and triggers increased cellular infiltration of the subarachnoid space [6].

The CNS can exclude circulating cells and harmful compounds from blood via the blood-brain barrier (BBB) and the blood-CSF barrier, which are formed by tight junction proteins around cerebral epithelial cells of the choroid plexus [7]. Claudins are integral membrane tight junction proteins localized at tight junctions and sufficient for the formation of tight junctions at cell-cell contacts [8]. Claudin 1, 2 and 5 were reported to be present in epithelial cells of the choroid plexus [9]. Also, claudin-5-deficient mice displayed size-selective loosening of the BBB and died shortly after birth [10]. In viral infection, brain microvascular endothelial cells exposed to the human immunodeficiency virus-1 (HIV-1) Tat protein decreased expression of claudin-5 and induced a redistribution of claudin-5 from cell-cell borders [11]. In bacterial infection, lipopolysaccharide can decrease expression of claudin-5 protein, resulting in increased permeability of rat brain microvascular endothelial cells [12]. In parasite infection, BBB impairment with enhanced expression of claudin-5 was reported in experimental cerebral toxocariasis [13].

In an attempt to characterize the mechanisms underlying *A. cantonensis*-induced blood-CSF barrier dysfunction, we investigated the association of NF-κB, MMP-9 and claudin-5 in the choroid plexus of the mouse brain after infection with *A. cantonensis*. The objective of the present study was to investigate alteration of claudin-5 via the NF-κB-MMP-9 pathway in mice with eosinophilic meningoencephalitis.

## Materials and Methods

### Experimental animals

Five-week-old male BALB/c mice were purchased from the National Laboratory Animal Center (Taipei, Taiwan) and maintained in a temperature-controlled environment with a 12 h light/12 h dark cycle and provided with Purina Laboratory Chow and water *ad libitum*. Mice were kept in a specific pathogen-free room at the Animal Center, Chung-Shan Medical University (Taichung, Taiwan) for more than one week before the experimental infection. All procedures were done in accord with the protocols approved by the Institutional Animal Care and Use Committee of Chung-Shan Medical University (Approval Number: 1087) and the institutional guidelines for animal experiments.

### Antibodies

Anti-mouse monoclonal antibodies IκB-α, p-IκB-α, NF-κB and p-NF-κB generated in rabbits were purchased from Cell Signaling Technology (Beverly, MA). Goat anti-mouse claudin-5 polyclonal antibody was purchased from Santa Cruz Biotechnology (CA, USA). Mouse anti-mouse β-actin monoclonal antibody was purchased from Sigma (St. Louis, MO, USA). Goat anti-mouse albumin polyclonal antibody was purchased from Bethyl Laboratories (Montgomer, USA). Horseradish peroxidase (HRP)-conjugated anti-rabbit IgG, HRP-conjugated anti-goat IgG and HRP-conjugated anti-mouse IgG were purchased from Jackson ImmunoResearch Laboratories (West Grove, PA, USA).

### Larval preparation

Infective larvae (L_3_) of *A. cantonensis* were obtained originally from wild giant African snails (*Achatina fulica*) that were propagated for several months and infected with *A. cantonensis* L_1_ by rats (the definitive host) at the Wufeng Experimental Farm (Taichung, Taiwan). The L_3_ larvae were recovered essentially as described [14] but with some modification. Briefly, snail shells were crushed and the tissues were homogenized in a pepsin-HCl solution (pH 1–2, 500 IU pepsin/g tissue) and digested with agitation at 37°C for 2 h. The larvae in the sediment were collected by serial washes in double-distilled water and counted under a microscope. The identity of the L_3_ larvae of *A. cantonensis* was confirmed as described [15].

### Animal infection

A total of 120 male BALB/c mice were randomly allocated to five experimental groups (D_5_, D_10_, D_15_, D_20_ and D_25_) and a control group of 20 mice each. Mice did not have access to food or water for 12 h before infection. Mice in the experimental groups D_5_, D_10_, D_15_, D_20_ and D_25_ were each infected with 50 *A. cantonensis* larvae by oral inoculation, and were sacrificed on days 5, 10, 15, 20 or 25 post-inoculation (PI), respectively. The control mice received only water and were sacrificed on day 25 PI. Brains were rapidly removed and frozen in liquid nitrogen.

### Treatment of animals

Forty mice were randomly allocated to four treatment groups (10 mice/group). Two groups of GM6001-treated mice were infected with 50 larvae and inhibited with specific MMPs inhibitor, GM6001 (2 mg/kg/day, Chemicon International, USA) on days 5 and 15 PI for seven consecutive days intraperitoneally, respectively. Mice were sacrificed on day 22 PI and the CSF was collected for claudin-5 analysis. Two groups of MG132-treated mice were infected with 50 larvae and treated with 1.5 or 3.0 mg/kg/day MG132 (Cayman Chemical, Ann Arbor, MI) for twenty consecutive days. Mice were sacrificed on day 22 PI and the brain was collected for NF-κB p-p65 and MMP-9 analysis.

### CSF collection

Mice were anesthesized by intraperitoneal urethane (1.25 g/kg) injection. Mouse placed in a stationary instrument with 135 degree from the head and body. Skin of neck shaved and swabbed with 70% ethanol (three times). Subcutaneous tissue and muscles were separated. Capillary tube through dura mater into citerna magna and CSF well inpoured capillary tube. Inject CSF into a 0.5 ml eppendorf tube and centrifuged at 3000×*g* at 4°C for 5 min. Collection of supernatant in a 0.5 ml eppendorf tube and kept at −80°C freezer.

### Western blot analysis

The mouse brains were homogenized in RIPA lysis buffer (150 mM sodium chloride, 1% (v/v) NP-40, 0.5% (v/v) deoxycholic acid, 0.1% (w/v) SDS and 50 mM Tris, pH 7.5) containing Protease Inhibitor Cocktail (Sigma, St. Louis, MO, USA). The homogenates were centrifuged at 12,000 *g* for 10 min at 4°C. Protein concentration was determined with protein assay kits (Bio-Rad, CA, USA) using bovine serum albumin (BSA) as the standard. Samples were mixed with an equal volume of loading buffer (62.5 mM Tris-HCl, pH 6.8, 10% (v/v) glycerol, 2% SDS, 5% (v/v) 2-mercaptoethanol and 0.05% (w/v) bromophenol blue) and heated for 5 min at 95°C. The mixture was subjected to SDS-PAGE and transferred electrophoretically to nitrocellulose membranes at a constant current of 190 mA for 90 min. Membranes were blocked with 5% (w/v) non-fat milk in PBS containing 0.1% (v/v) Tween 20 (PBST) for 1 h at room temperature. Membranes were reacted with primary antibodies at 37°C for 1 h. Membranes were washed three times with PBST, HRP-conjugated secondary antibody (1∶10,000 dilution) was added and incubated at 37°C for 1 h to detect primary bound antibody. Reactive proteins were detected by enhanced chemiluminescence (Amersham, Little Chalfont, Bucks, UK) and the density of specific immunoreactive bands was quantified by densitometric scanning.

### Gelatin zymography

Brain homogenates were subjected to SDS-PAGE (7.5% (w/v) polyacrylamide gel containing 0.1% (w/v) gelatin (Sigma, St. Louis, MO, USA) in running buffer (25 mM Tris, 250 mM glycine, 1% SDS) at room temperature. The gel was washed twice in double-distilled water containing 2.5% Triton X-100 for 30 min each time, then incubated in reaction buffer (50 mM Tris-HCl, pH 7.5, 200 mM NaCl, 10 mM CaCl_2_, 0.02% (w/v) Brij®-35, 0.01% (w/v) NaN_3_) at 37°C for 18 h. The gel was stained with 0.25% (w/v) Coomassie brilliant blue R-250 (Sigma, St. Louis, MO, USA) for 1 h and then destained in 15% (v/v) methanol, 7.5% (v/v) acetic acid. Gelatinase activity was detected as unstained bands on a blue background. Quantitative analysis was done with a computer-assisted imaging densitometer system (UN-SCAN-IT™ gel version 5.1, Silk Scientific, UT).

### Eosinophil counts in the CSF

The CSF was collected and centrifuged at 400 *g* for 10 min, the sediment was recovered and mixed gently with 100 µL of Unopette buffer (Vacutainer System; Becton Dickinson, Franklin Lakes, NJ) and 2 µL of acetic acid, and placed into the cell counting chamber of a hemocytometer (Paul Marienfeld, Lauda-Koenigshofen, Germany) to count the eosinophils.

### Assay of blood-CSF barrier permeability

Blood-CSF barrier permeability was evaluated by assessing the concentration of Evans blue in CSF. At 2 h before sacrifice, mice were injected with 2% Evans blue (100 mg/kg body weight; Sigma, St. Louis, MO, USA) in saline into a tail vein. The average concentration of Evans blue in CSF was calculated from measurement of the absorbance at 620 nm in a spectrophotometer (Hitachi U3000; Tokyo, Japan).

### Statistical analysis

Results for the different groups were compared with the non-parametric Kruskal-Wallis test followed by Dunn's multiple comparison of means. All results are presented as mean ± standard deviation (S.D.). Statistically significant difference was set at *P*<0.05.

## Results

### Kinetic studies for p-IκB-α and p-NF-κB in mouse brain

**Figure 1:**
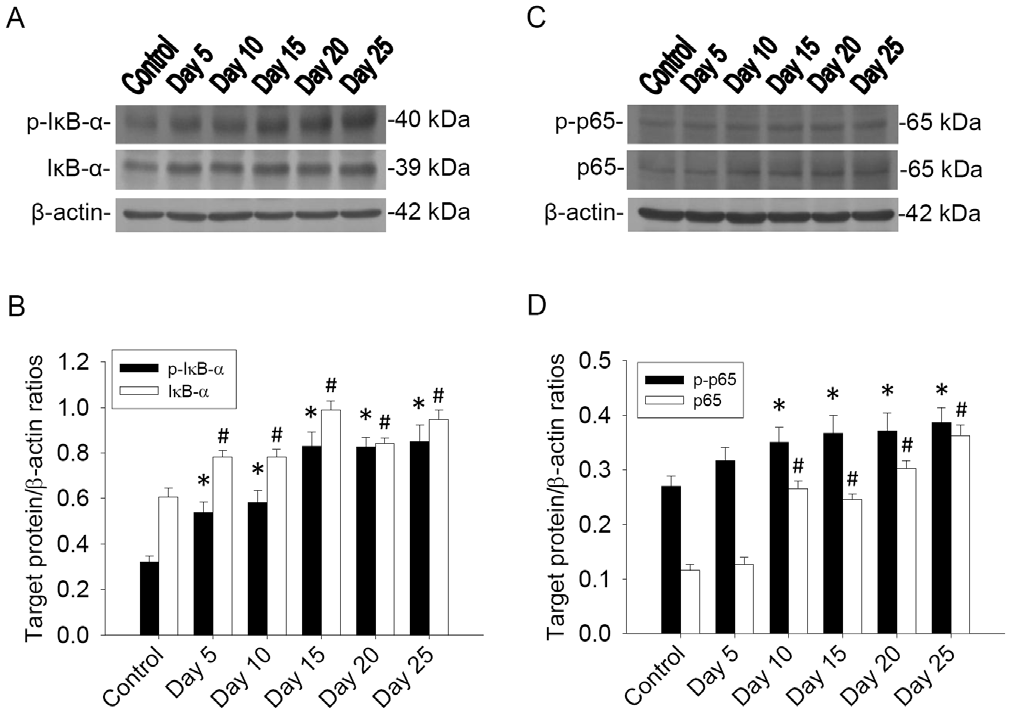
Time-course studies for p-IκB-α and p-NF-κB p65 in mouse brain (A and C). Protein bands of p-IκB-α, IκB-α, p-NF-κB and NF-κB from the brain of mice (n = 5) infected with *A. cantonensis*. β-actin was used as a loading control. (B and D) Quantification and normalization to β-actin showed significant increases not only in p-IκB-α and IκB-α (*P<0.05) but also in p-NF-κB p65 and NF-κB p65 (^#^ *P*<0.05). Bars represent mean ± S.D. from three independent experiments performed in duplicate.

p-IκB-α, IκB-α, p-NF-κB and NF-κB in the brain of mice infected with *A. cantonensis* were analyzed by western blotting. p-IκB-α and IκB-α in infected mouse brains were elevated significantly on days 5, 10, 15, 20 and 25 PI compared to the levels in the uninfected mice brains. Additionally, p-NF-κB and NF-κB proteins were increased significantly on days 10, 15, 20 and 25 PI in infected mice brains compared to the levels in uninfected mice brains (Figure 1).

### Kinetic studies for claudin-5 in mouse brain and CSF

**Figure 2:**
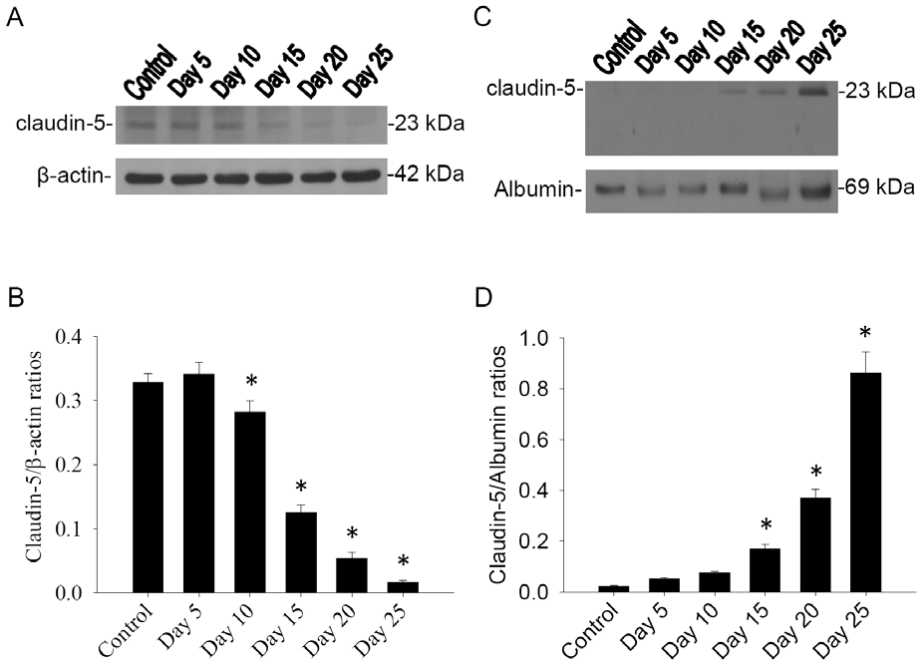
Time-course studies for claudin-5 in mouse brain and CSF. Protein bands of (A) brain claudin-5 and (C) CSF claudin-5 from mice (n = 5) infected with *A. cantonensis*. β-actin and albumin were used as loading control. (B and D) Quantification and normalization to loading control of claudin-5 showed significant decrease (*P<0.05) in brain but significant increase in CSF (*P<0.05). Bars represent mean ± S.D. from three independent experiments performed in duplicate.

Detection of claudin-5 protein levels in the brains and CSF by western blotting. Quantitative analysis of claudin-5 was performed with a computer-assisted imaging densitometer system. Densitometric scanning quantification of mice expressed as the ratio of the band density of claudin-5 to that of β-actin or albumin at each experimental group. Claudin-5 in the brains were significantly decreased (*P*<0.05) on days 10, 15 and 20 PI of infected mice than uninfected mice. However, claudin-5 in CSF was significantly increased (*P*<0.05) on days 10, 15 and 20 PI of infected mice than uninfected mice (Figure 2).

### Influence of treatment with MG132 and GM6001

**Figure 3:**
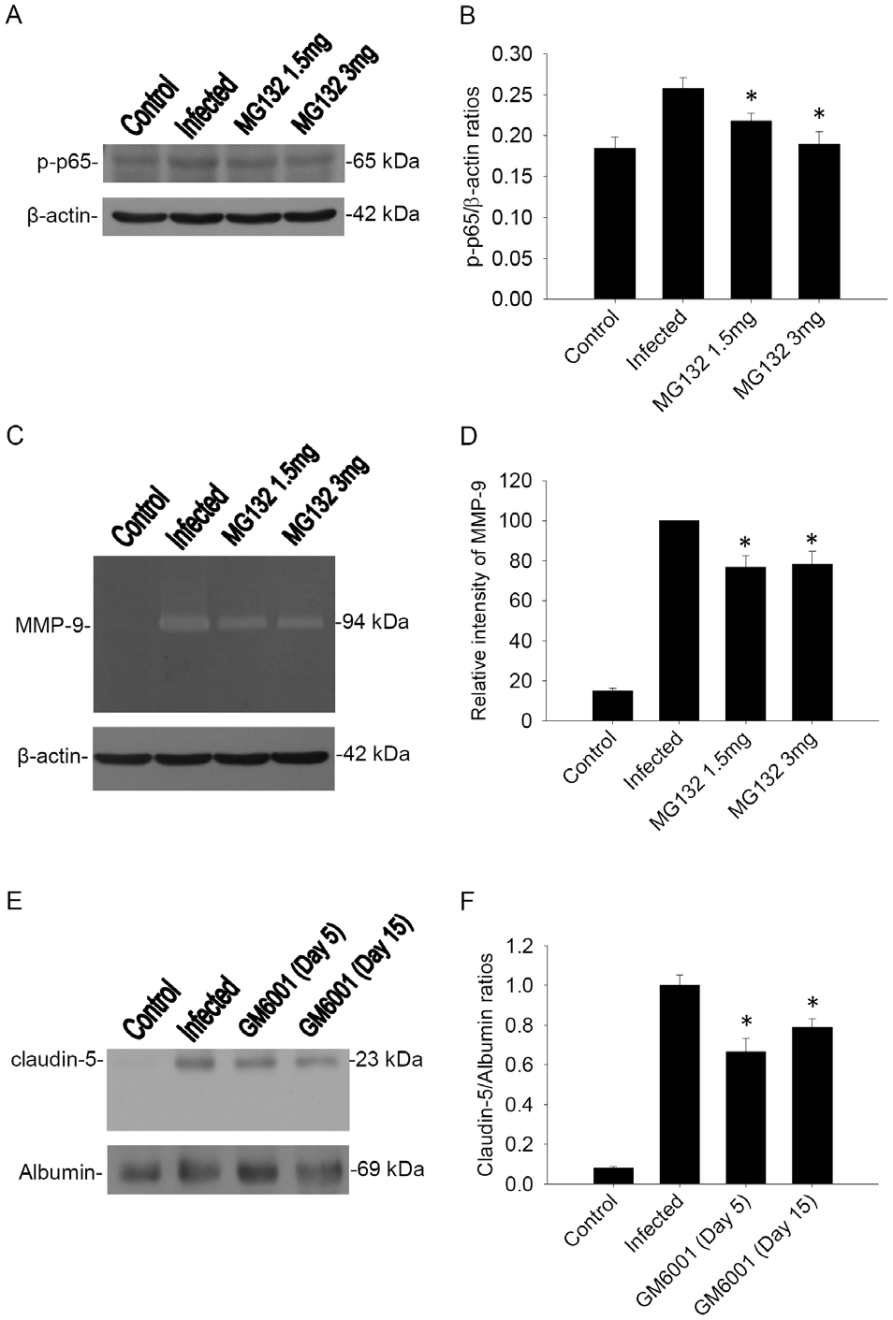
Influence of treatment with MG132 and GM6001 after infection. The test groups were: uninfected mice (control), n = 5; *A. cantonensis*-infected untreated mice (infected), n = 5; mice treated with 1.5 mg of MG132, n = 5; mice treated with 3 mg of MG132, n = 5; mice treated with GM6001 on day 5 PI, n = 5; and mice treated with GM6001 on day 15 PI, n = 5. (A) The p-NF-κB p65 bands were analyzed by western blotting using specific antibody from the brains. (B) Ratios of p-NF-κB p65/β-actin showed significant decrease (*P<0.05) in MG132 in treated mice versus infected mice. (C) The MMP-9 activities were analyzed by gelatin zymography from the brains. (D) The intensities of MMP-9 were decreased significantly (*P<0.05) in MG132-treated mice versus infected mice. (E) The claudin-5 bands were analyzed by western blotting using specific antibody from the CSF. (F) Ratios of claudin-5 were decreased significantly (*P<0.05) in GM6001-treated mice versus infected mice. Bars represent mean ± S.D. from three independent experiments performed in duplicate.

The effect of MG132 was investigated in a murine angiostrongyliasis model. MG132 binds pocket in the active site of proteasome and attenuates degradation of p-IκB-α to decrease phosphorylation of NF-κB. Western blotting measured the levels of protein p-p65, which was increased significantly in the brain of infected mice compared to the level in uninfected controls. The levels of protein p-p65 were reduced significantly after treatment with MG132. Additionally, gelatin zymography demonstrated the activity of MMP-9, which was elevated significantly in the brain of infected mice compared to the level in uninfected controls. The activity of MMP-9 was decreased significantly after treatment with MG132. Western blotting measured the level of claudin-5 protein, which was increased significantly in CSF of infected mice compared to the level in uninfected controls. The levels of claudin-5 protein were reduced significantly after treatment with GM6001 in a murine angiostrongyliasis model. Quantitative analysis of p-p65, MMP-9 and claudin-5 were performed with a computer-assisted imaging densitometer system. Densitometric scanning quantification of mice expressed as the ratio of the band density of p-p65, MMP-9 or claudin-5 to that of β-actin or albumin at each experimental group (Figure 3).

### Correlation between claudin-5 and Evans blue

**Figure 4:**
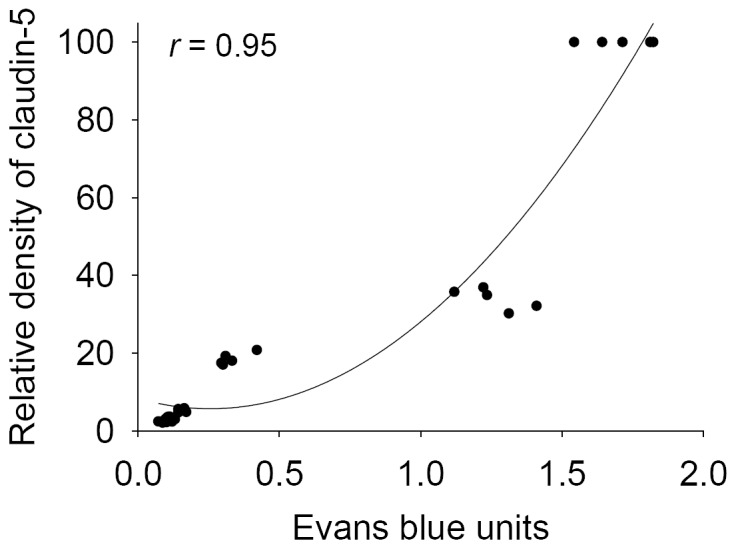
Correlation between claudin-5 and Evans blue. The claudin-5 levels were significantly correlated (*r* = 0.95; *P*<0.05) with Evans blue.

Evans blue is normally excluded from CSF by an intact blood-CSF barrier but it can enter CSF when the integrity of the blood-CSF barrier is disrupted. This method is often used to assess simple alterations in the blood-CSF barrier. The blood-CSF barrier permeability assays for the mouse CSF after infection with *A. cantonensis* showed a significant correlation with the degradation of claudin-5 (Figure 4).

### Correlation of claudin-5 with CSF eosinophils

**Figure 5:**
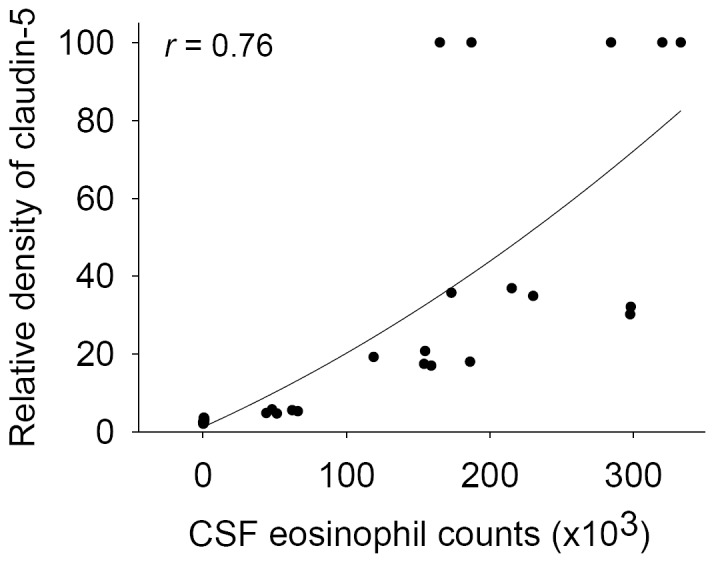
Correlation of claudin-5 with CSF eosinophils. The level of claudin-5 was correlated significantly (*r* = 0.76; *P*<0.05) with CSF eosinophil count, using Spearman's ranking correlation test.

CSF eosinophilia was found only in infected mice. The level of claudin-5 was correlated significantly with the eosinophil count in CSF (Figure 5).

## Discussion

Infection of mice with *A. cantonensis* causes brain injury and increased expression of NF-κB in the brains of ICR [16] and BALB/c strains of mice [17]. Exposure of human bone marrow endothelial cells to *Trypanosoma brucei gambiense* induced NF-κB translocation to the nucleus and CNS barrier dysfunction [18]. In addition, over-expression of IκB-α completely blocked NF-κB binding to the MMP-9 promoter, demonstrating that NF-κB activity mediated expression of MMP-9 in primary rabbit and human dermal fibroblasts [19]. In the present study, significant elevation of p-IκB-α and p-NF-κB was demonstrated in brain samples from mice with eosinophilic meningoencephalitis caused by infection with *A. cantonensis*. Mice treated with MG132 had decreased NF-κB phosphorylation and MMP-9 production in eosinophilic meningoencephalitis. These results demonstrate the correlation between NF-κB phosphorylation and MMP-9 activity during infection with *A. cantonensis*.

HIV-1 envelope glycoprotein gp120 can reduce BBB integrity and disrupt tight junction proteins (occludin and claudin-5) by MMP-9 in brain vascular endothelial cells [20], [21]. Also, amyloid-β decreases expression of claudin-5, increases activities of MMP-2, -9, causes BBB leakage and promotes BBB permeability in humans with cerebral amyloid angiopathy [22]. Treatment with MMP-9 chelator GM6001 can reverse disruption of claudin-5 and BBB permeability in brain edema of mice with acute liver failure [23]. Additionally, MMPs contributed to the inflammatory breakdown of the blood-CSF barrier in porcine choroids plexus epithelial cells [24]. MMP-12 might have an important role in the degradation of elastin and participate in the breakdown of blood-CSF barrier in mice with eosinophilic meningoencephalitis caused by *A. cantonensis* [25]. These studies demonstrated that MMPs might be associated with the disruption of brain barrier during inflammation. In this study, we showed that the degradation of claudin-5 is correlated significantly with blood-CSF barrier permeability. In addition, the degradation of claudin-5 was reduced significantly when mice were treated with GM6001 and *A. cantonensis-*induced disruption of blood-CSF barrier was prevented by treatment with this MMP inhibitor. These results demonstrate that *A. cantonensis* can activate MMP-9 and leukocyte-derived MMP-9 can contribute to tight junction damage and impairment of the blood-CSF barrier.

Tight junctions control the paracellular permeability of epithelial cells and dysregulated permeability is associated with inflammatory diseases. In a cell model for blood-CSF barrier consisting of porcine choroid plexus epithelial cells infected with *Streptococcus suis*, which causes bacterial meningitis, the bacteria induced loss of blood-CSF barrier function and might facilitate trafficking of leukocytes across the barrier [26], [27]. There are two possible routes to explain a traversal mechanism during bacterial meningitis: (1) leukocyte infiltration-directed transcellular migration; and (2) leukocytes crossing the barrier by paracellular migration [27]. In this study, we demonstrated that claudin-5 was decreased in brain and increased in CSF of mice with angiostrongyliasis meningoencephalitis. Reduction of claudin-5 implies increased blood-CSF barrier permeability and leukocyte migration across the blood-CSF barrier in choroid plexus after infection with *A. cantonensis*. We suggest that leukocytes migrate across the blood-CSF barrier via the paracellular route and subsequently reach inflammatory space in mice infected with *A. cantonensis*. Therefore, the traversal mechanism of leukocytes across blood-CSF barrier could depend on the type of pathogen.

**Figure 6:**
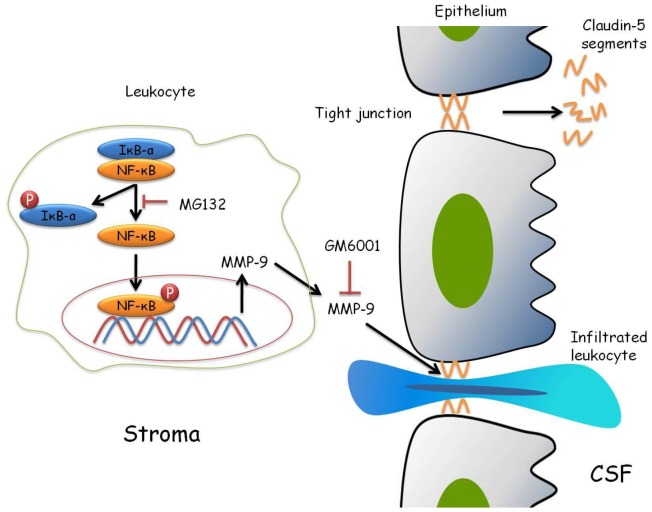
Possible mechanisms of matrix metalloproteinase (MMP)-9 leading to claudin-5 degradation via the NF-κB pathway. The activation of IκB-α and NF-κB could upregulate MMP-9 production in *A. cantonensis*-induced leukocytes. Blocking IκB-α and NF-κB signaling by MG132 could reduce MMP-9 production. Further, blocking MMP-9 activity by GM6001 could reduce claudin-5 degradation and blood-CSF barrier permeability during angiostrongyliasis meningoencephalitis. Therefore, we suggest that MMP-9 could cause claudin-5 degradation and promote leukocyte infiltration into CSF by the paracellular route during *A. cantonensis* infection in mouse choroid plexus.

The claudin family, integral components of tight junctions, are responsible for determining the permeability of paracellular transport within epithelial cell layers. The present study showed that MMP-9 might contribute to the degradation of claudin-5 in the blood-CSF barrier. MMP-9 production was correlated with claudin-5 degradation and blood-CSF barrier permeability in angiostrongyliasis meningoencephalitis. Therefore, we propose a possible mechanism (Fig. 6) to explain the contribution of MMP-9 to blood-CSF barrier permeability. In mice infected with *A. cantonensis*, the parasite-induced eosinophilia and inflammation might lead to the induction of the phosphorylation levels of IκB-α and NF-κB. The activation of IκB-α and NF-κB could upregulate MMP-9 production. Blocking IκB-α and NF-κB signaling by MG132 could reduce MMP-9 production. Further, blocking MMP-9 activity by GM6001 could reduce claudin-5 degradation and blood-CSF barrier permeability during angiostrongyliasis meningoencephalitis. MMP-9 could cause claudin-5 degradation and promote leukocyte infiltration into CSF via the paracellular route during infection with *A. cantonensis* in mouse choroid plexus.

In summary, increased blood-CSF barrier permeability is associated with disruption of tight junction proteins elicited by activation of MMP-9. Claudin-5 degradation and blood-CSF barrier dysfunction in brain with angiostrongyliasis is mediated by MMP-9 *via* the IκB-α/NF-κB/MMP-9 signaling pathway. These mechanistic insights could be used for the pathophysiologic evaluation of blood-CSF barrier breakdown and provide the basis for therapeutic strategies for *A. cantonensis*-induced tight junction disruption.
